# Hypertension Pharmacological Treatment in Adults: A World Health Organization Guideline Executive Summary

**DOI:** 10.1161/HYPERTENSIONAHA.121.18192

**Published:** 2021-11-15

**Authors:** Akram Al-Makki, Donald DiPette, Paul K. Whelton, M. Hassan Murad, Reem A. Mustafa, Shrish Acharya, Hind Mamoun Beheiry, Beatriz Champagne, Kenneth Connell, Marie Therese Cooney, Nnenna Ezeigwe, Thomas Andrew Gaziano, Agaba Gidio, Patricio Lopez-Jaramillo, Unab I. Khan, Vindya Kumarapeli, Andrew E. Moran, Margaret Mswema Silwimba, Brian Rayner, Apichard Sukonthasan, Jing Yu, Nizal Saraffzadegan, K. Srinath Reddy, Taskeen Khan

**Affiliations:** Indiana University Health Arnett, Lafayette (A.A.-M.).; Indiana University School of Medicine – West Lafayette (A.A.-M.).; College of Pharmacy, Purdue University, West Lafayette, IN (A.A.-M.).; Department of Medicine, University of South Carolina School of Medicine, University of South Carolina, Columbia (D.D.).; Department of Epidemiology, School of Public Health and Tropical Medicine, Tulane University, New Orleans, LA (P.K.W.).; Evidence-based Practice Center and Robert D. and Patricia E. Kern Center for the Science of Health Care Delivery, Mayo Clinic, Rochester, MN (M.H.M.).; Department of Internal Medicine, Division of Nephrology and Hypertension, University of Kansas Medical Center, Kansas City (R.A.M.).; Department of Health Research Methods, Evidence and Impact, McMaster University, Hamilton, ON, Canada (R.A.M.).; Department of Internal Medicine, Colonial War Memorial Hospital and National Medicine and Therapeutics Committee, Ministry of Health, Fiji (S.A.).; Faculty of Nursing Sciences, International University of Africa (IUA), Khartoum, Sudan (H.M.B.).; Coalition for Americas’ Health/Coalición América Saludable CLAS, representing civil society organizations in Latin America, Dallas, TX (B.C.).; Faculty of Medical Sciences, The University of the West Indies, Cave Hill Campus, St. Michael, Barbados (K.C.).; St. Vincent’s University Hospital & University College Dublin, Ireland (M.T.C.).; Federal Ministry of Health, Abuja, Nigeria (N.E.).; Harvard Medical School, Boston, MA (T.A.G.).; Brigham and Women’s Hospital, Boston, MA (T.A.G.).; Mulago National Referral Hospital, Kampala, Uganda (A.G.).; Masira Research Institute, Medical School, Universidad de Santander, Bucaramanga, Colombia (P.L.-J.).; Department of Family Medicine, The Aga Khan University, Pakistan (U.I.K.).; Directorate of Non-Communicable Diseases, Ministry of Health, Colombo, Sri Lanka (V.K.).; Global Hypertension Control, Resolve to Save Lives, an initiative of Vital Strategies, NY (A.E.M.).; Division of General Medicine, Columbia University Irving Medical Centre, NY (A.E.M.).; University Teaching Hospital (Adult), Lusaka, Zambia and Faculty of Pharmacy, Lusaka Apex Medical University (M.M.S.).; Division of Nephrology and Hypertension, Groote Schuur Hospital and University of Cape Town, South Africa (B.R.).; Department of Medicine, Bangkok Hospital Chiang Mai, Mueang Chiang Mai, Thailand (A.S.).; Hypertension Center, Department of Cardiology, Lanzhou University Second Hospital, China (J.Y.).; Isfahan Cardiovascular Research Center, Cardiovascular Research Institute, Isfahan University of Medical Sciences, Iran (N.S.).; School of Population and Public Health, Faculty of Medicine, University of British Columbia, Vancouver, Canada (N.S.).; Public Health Foundation of India, New Delhi (K.S.R.).; Department of Non-Communicable Diseases, World Health Organization, Geneva, Switzerland (T.K.).; Department of Public Health Medicine, University of Pretoria, Gauteng, South Africa (T.K.).

**Keywords:** blood pressure, cardiovascular disease, hypertension, pharmacotherapy

## Abstract

Hypertension is a major cause of cardiovascular disease and deaths worldwide especially in low- and middle-income countries. Despite the availability of safe, well-tolerated, and cost-effective blood pressure (BP)-lowering therapies, <14% of adults with hypertension have BP controlled to a systolic/diastolic BP <140/90 mm Hg. We report new hypertension treatment guidelines, developed in accordance with the World Health Organization Handbook for Guideline Development. Overviews of reviews of the evidence were conducted and summary tables were developed according to the Grading of Recommendations, Assessment, Development, and Evaluations approach. In these guidelines, the World Health Organization provides the most current and relevant evidence-based guidance for the pharmacological treatment of nonpregnant adults with hypertension. The recommendations pertain to adults with an accurate diagnosis of hypertension who have already received lifestyle modification counseling. The guidelines recommend BP threshold to initiate pharmacological therapy, BP treatment targets, intervals for follow-up visits, and best use of health care workers in the management of hypertension. The guidelines provide guidance for choice of monotherapy or dual therapy, treatment with single pill combination medications, and use of treatment algorithms for hypertension management. Strength of the recommendations was guided by the quality of the underlying evidence; the tradeoffs between desirable and undesirable effects; patient’s values, resource considerations and cost-effectiveness; health equity; acceptability, and feasibility consideration of different treatment options. The goal of the guideline is to facilitate standard approaches to pharmacological treatment and management of hypertension which, if widely implemented, will increase the hypertension control rate world-wide.

Cardiovascular disease (CVD) is the leading cause of death worldwide, with most deaths attributed to hypertension resulting from coronary heart disease or stroke and more than three quarters of these deaths occurring in low- and middle-income countries.^1^ The level of high blood pressure (BP) is directly related to many adverse outcomes, including CVD and kidney disease.^2^ Conversely, clinical trials have repeatedly demonstrated that lowering BP in adults with high baseline BP level provides an effective means for preventing CVD. Definitions of hypertension are useful for clinical decision-making and are typically based on CVD risk and level of BP at which antihypertensive medication is effective for preventing CVD.^3^ A common definition of hypertension is based on an average systolic BP (SBP) ≥140 mm Hg, diastolic BP (DBP) ≥90 mm Hg, or self-reported use of antihypertensive medication. Using this definition, it has been estimated that ≈1.4 billion adults have hypertension, worldwide, but <14% have their BP controlled with antihypertensive drug therapy to an SBP/DBP <140/90 mm Hg (<8% in low- and middle-income countries).^3^ Even more disturbing is the recent trend in a high-income country (the United States), where rate of hypertension control to an SBP/DBP <140/90 mm Hg reached a high of close to 54% in 2013 to 2014, but dramatically eroded to <44% in 2017 to 2018.^4^ Given this decrease, the Surgeon General of the United States issued a report for a “Call to Action to Control Hypertension” making the control of hypertension a national priority.^5^

In 1978, the World Health Organization (WHO) published one of the earliest clinical practice guidelines for the diagnosis and management of arterial hypertension, which were later updated in 1999 and 2003.^6,7^ In 2007, the WHO published some recommendations for the management of hypertension in guidelines for the assessment and management of total CVD risk. However, these are now outdated considering new evidence and practices.^8^ Guidance is particularly needed now on some controversial issues, such as the threshold level of BP at which to start pharmacological treatment and whether laboratory testing and CVD risk assessment are needed before initiating antihypertensive pharmacological therapy. In the past decade, the WHO included diagnosis and management of hypertension in a total CVD risk approach as part of the WHO Package of Essential Noncommunicable Disease Interventions (WHO PEN) 2007, 2010, and 2013.^9^ However, this approach preceded recent advances in the pharmacological management of hypertension. More recently, the WHO has provided information about the diagnosis and management of hypertension in the Global HEARTS Initiative including the HEARTS Technical Package and the HEARTS in the Americas Program.^10,11^ However, these too provide general practical information and not specific guidelines and recommendations. The WHO Essential Medicines List includes ACE (angiotensin-converting enzyme) inhibitors, calcium channel blockers, angiotensin receptor blockers, and thiazide diuretics for management of hypertension. In June 2019, single-pill combination antihypertensive medications were added to the WHO Essential Medicines List.^12^

Herein, we summarize the recommendations of the 2021 WHO Guidelines for the Pharmacological Treatment of Hypertension in Adults. They include guidance on the BP threshold for the initiation of pharmacological treatment for hypertension, initial and longer-term visit intervals for follow-up of treated patients, treatment target BP levels, and the best use of health care workers for management of hypertension. The 2021 WHO hypertension guidelines aim to provide the most current and relevant evidence-based guidance for pharmacological treatment of hypertension in nonpregnant adults, with a particular focus on practice in middle- and low-income countries.

Although these guidelines do not address modifiable risk factors for hypertension such as unhealthy diet, overweight, obesity, physical inactivity, or consumption of alcohol, a comprehensive treatment plan for hypertension should address these risk factors through lifestyle modifications and other interventions.^3^ Likewise, treatment of hypertension should be accompanied by management of other CVD risk factors such as cigarette smoking, diabetes, lipid abnormalities, and comorbid conditions.^13^

The 2021 WHO hypertension guidelines are focused on management in routine, primary care settings (primary care health care providers, family physicians, cardiologists, nephrologists, and any providers who manage hypertension) and do not address the treatment of hypertensive emergencies or urgencies, secondary forms of hypertension, or resistant hypertension.

## Materials and Methods

### Guideline Contributors

To develop the hypertension guidelines, the WHO established 4 groups:

1. An internal WHO Steering Group to coordinate the guideline development process.
2. A Guideline Development Group (GDG), composed of a diverse group of primary care and subspecialty physicians who were hypertension experts, pharmacists, nurses, health-oriented academics, a patient representative, and policymakers to review the evidence and develop recommendations. The WHO selected the members of the GDG based on relevant expertise but also considered appropriate representation by region and sex. An independent methodologist facilitated GDG deliberations.
3. An External Review Group, composed of technical experts, representatives of hypertension patient groups, and ministries of health from low-resource countries, to provide peer review of the guidelines.
4. An independent contracted systematic review group that conducted the overview of reviews and summarized evidence.

The guidelines were developed in accordance with the WHO Handbook for Guideline Development. In brief, the WHO Steering Group, in collaboration with the Guideline Development Group, developed key questions and rated outcomes to identify those critical for the development of the guidelines. Conflicts of interest were handled in line with the current Compliance, Risk Management and Ethics policy and all members of the GDG were asked to fill in the standard WHO Declaration of Interest forms, which were reviewed. An overview of systematic reviews of the evidence were used to build summary of findings tables according to the Grading of recommendations, Assessment, Development and Evaluations (GRADE) approach. The Guideline Development Group developed recommendations, considering the certainty of the evidence, the balance between desirable and undesirable effects, resource requirements and cost-effectiveness, health equity, acceptability, patient values and preferences, and feasibility.

### Guideline Scope and Questions

**Figure 1.**
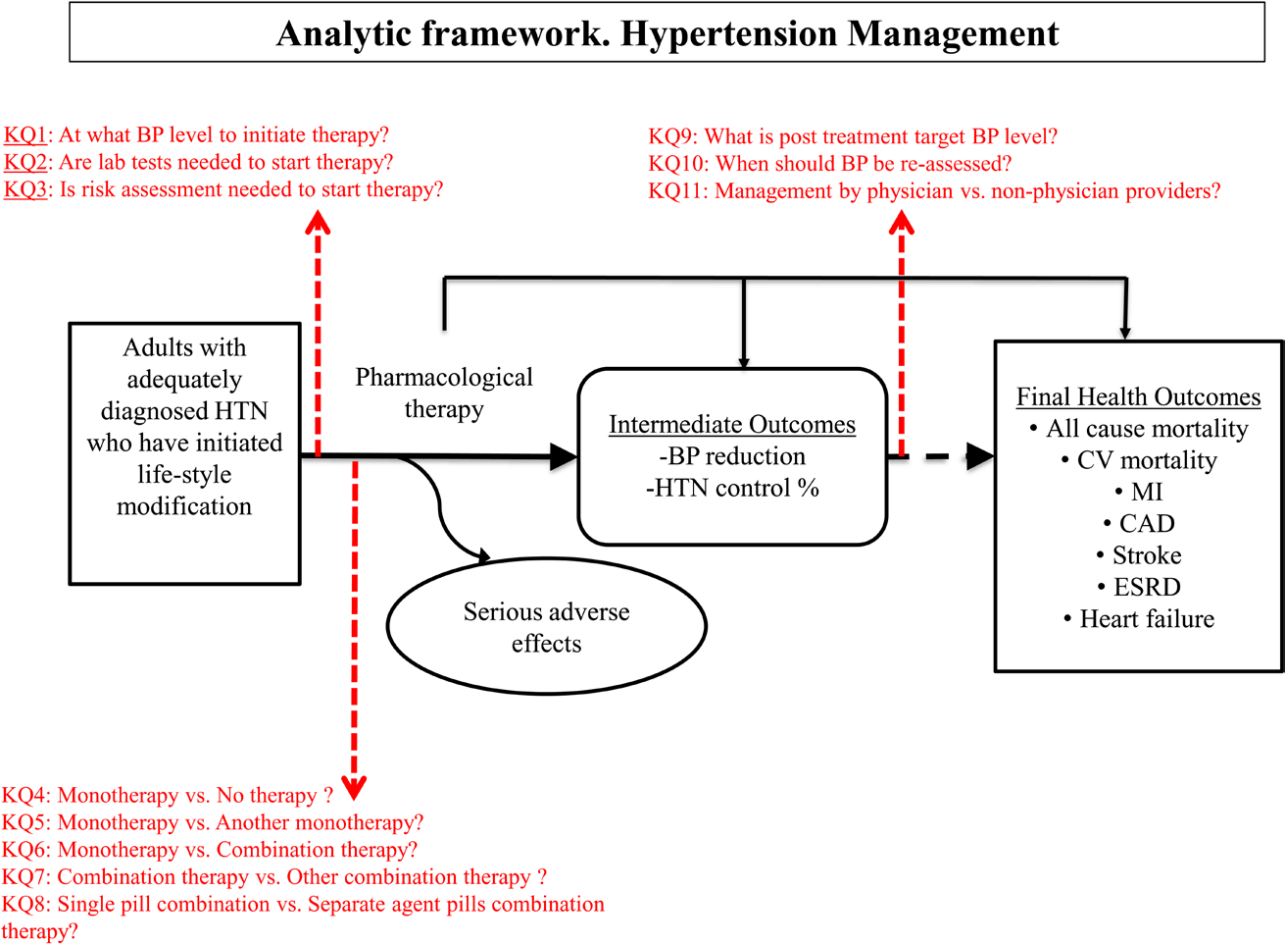
**Analytic framework for management of hypertension.** BP indicates blood pressure; CAD, coronary artery disease; CV, cardiovascular; ESRD, end-stage renal disease; HTN, hypertension; KQ, key question, and MI, myocardial infarction.

Members of the WHO GDG developed an analytic framework that linked hypertension treatment to important health outcomes (Figure 1) in which 11 clinical questions were prioritized. The framework linked pharmacological treatments of hypertension to intermediate health outcomes and final health outcomes, as well as adverse events of testing. The GDG rated each outcome on a scale from 1 to 9 and indicated whether it considered each outcome critical (rated 7–9), important (rated 4–6) or not important (rated 1–3), for decision-making.

### Reviews of Evidence

The systematic review group conducted systematic searches of the literature to identify existing systematic reviews published between 2015 and 2020 in PubMed, Embase, The Cochrane Library, and Epistemonikos that addressed the 11 questions. When multiple reviews were published, systematic reviews were prioritized to inform the recommendations based on quality (assessed by the Methodological Quality of Systematic Reviews, AMSTAR); how well they addressed the questions; whether they reported sufficient information to assess the quality of the evidence; whether they reported evidence for subgroups of interest (eg, patients with diabetes, CVD, chronic kidney disease [CKD], etc); and the date of the most recent search. Content experts were queried for additional literature. The reviews published since 2015 include the major studies that were published before 2015 and the more recently published landmark studies. We have carefully reviewed the studies that informed recent guidelines, and we reference them in the detailed guideline report. In addition to our primary search of the literature, we cross referenced existing systematic reviews and guidelines to identify any additional studies. All this information is detailed in the full guideline document and the Supplemental Material.

**Figure 2.**
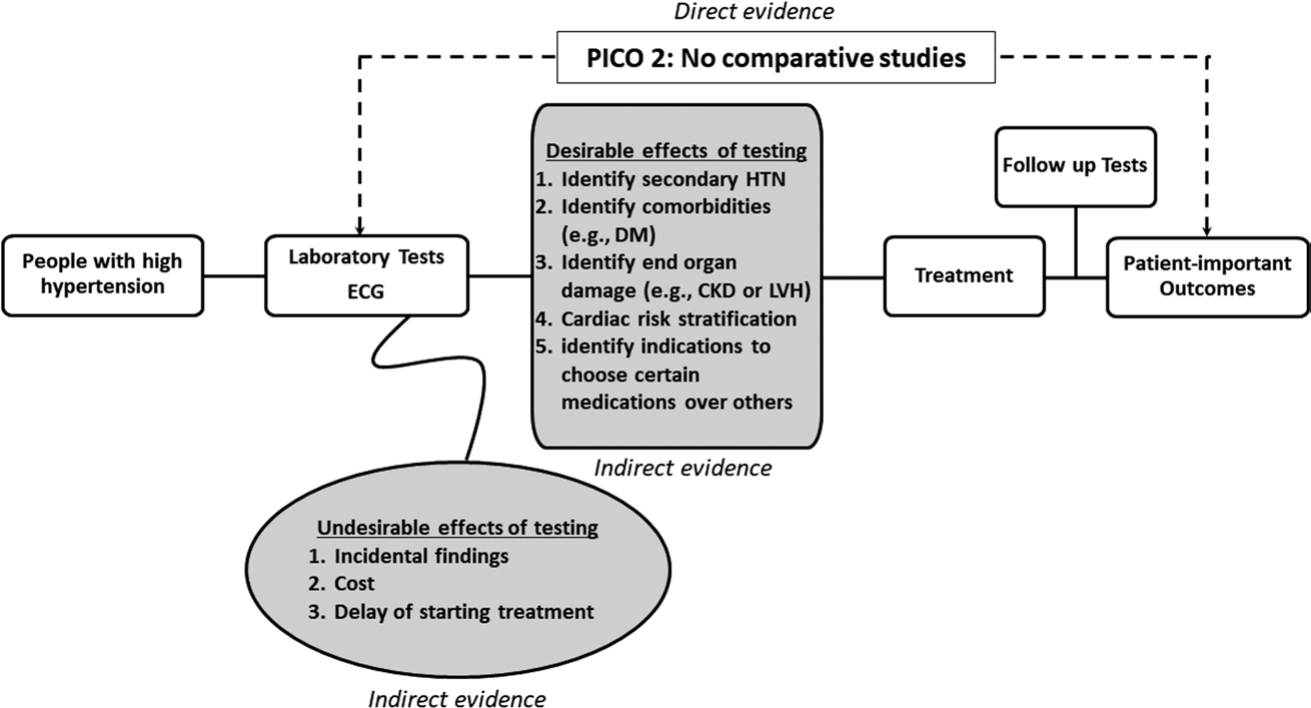
**Conceptual model used to derive indirect evidence that helped to answer the question about laboratory testing.** CKD indicates chronic kidney disease; DM, diabetes; HTN, hypertension; LVH, left ventricular hypertrophy; and PICO, population, intervention, comparison, and outcome.

A total of 159 systematic reviews and 17 additional primary studies were identified, including 9 individual patient data analyses. An additional overview of systematic reviews was used to inform other decision criteria in the evidence-to-decision framework, including patient’s values, resources, acceptability, feasibility, and equity. No direct evidence was identified for the question about obtaining laboratory testing. Therefore, a conceptual model was used to derive indirect evidence that could help to answer the question (Figure 2). The model linked testing to final health outcomes by describing the desirable and undesirable effects of testing. Indirect evidence incorporated in this model provided a rationale for testing and facilitated GDG recommendations.

### Certainty of Evidence and Strength of Recommendations

The GDG rated the certainty of evidence and developed the recommendations using the GRADE approach.^14^ When making recommendations, GRADE defines the quality of a body of evidence as “the extent of our confidence that the estimates of an effect are adequate to support a particular decision or recommendation”.^15^ The strength of the recommendations reflects the degree of confidence of the GDG that the desirable effects (eg, beneficial health outcomes) of the recommendations outweigh the undesirable effects (eg, adverse effects). According to the GRADE approach, the certainty of the evidence can be high, moderate, low, or very low (Table). The recommendations were graded into 2 categories:

**Table 1.**
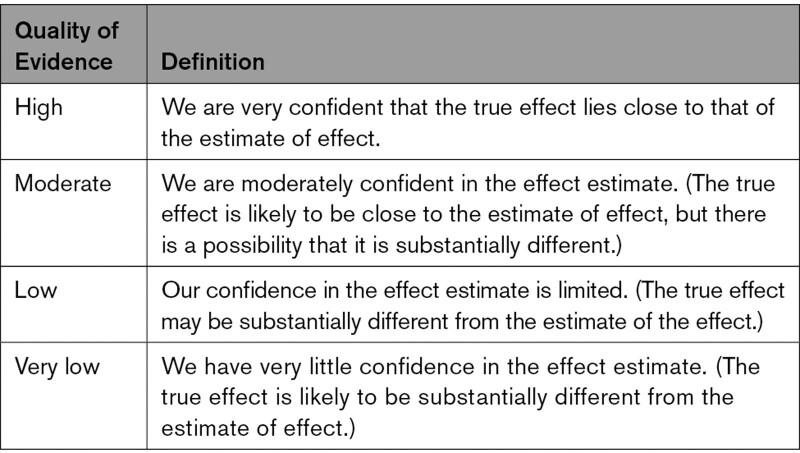
Quality of evidence and its implications

1. A strong recommendation represented GDG confidence that the desirable effects of adhering to the recommendation outweighed undesirable effects.
2. A weak or conditional recommendation represented a GDG conclusion that the desirable effects of adhering to the recommendation probably outweighed the undesirable effects.

### Deciding Upon Recommendations

The GDG initially met in person in July 2019 where they reviewed a scoping document and developed the specific Population, Intervention, Comparison, and Outcome questions to be addressed in the guidelines. Subsequently and because of the coronavirus disease 2019 (COVID-19) pandemic, the GDG met virtually for 4 sessions in February 2021. Systematic reviews and GRADE tables were discussed at the meeting. Formulation of recommendations and their strength were facilitated by the GDG chair and supported by the methodologist, with the aim of achieving unanimous consensus. While the plan was to use a simple majority vote, full consensus was reached on all recommendations.

## Results/Recommendations

**Figure 3.**
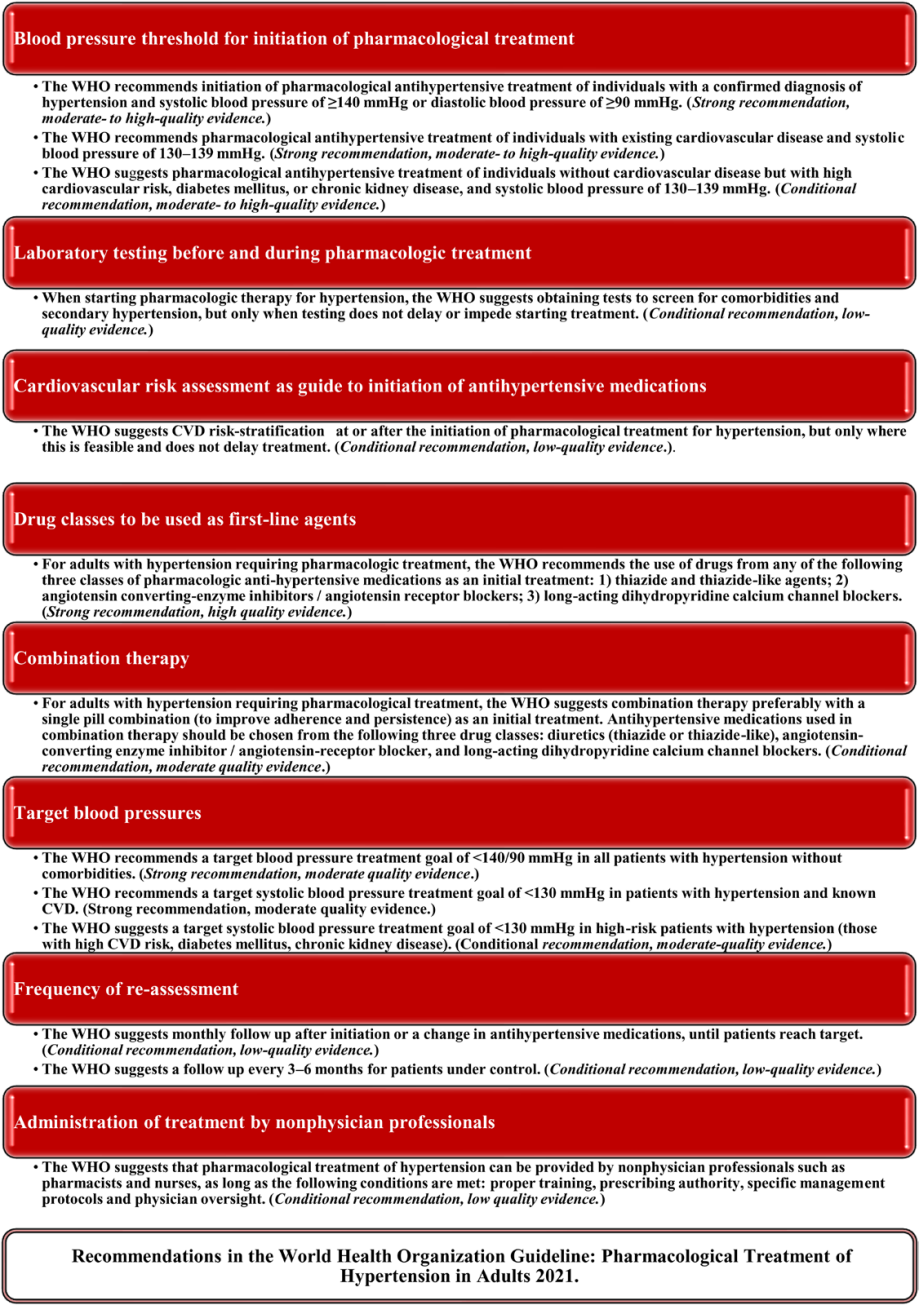
**Recommendations in the World Health Organization Guideline: Pharmacological Treatment of Hypertension in Adults 2021.** CVD indicates cardiovascular disease; and WHO, World Health Organization.

The 11 questions led to 8 recommendations that are summarized in Figure 3. GDG judgments on certainty of evidence, each decisional factor, contextual information, and balance of desirable and undesirable effects are available in the full WHO guideline. Each recommendation was accompanied by implementation remarks and considerations to facilitate adaptation and application by various countries and systems. The External Review Group provided peer review of the guidelines and ensured recommendations are aligned with current global needs. Evidence informing each of the recommendations, certainty of evidence, patients’ values, resources, feasibility, acceptability, and equity considerations can be accessed in the WHO’s full document that is available online (https://apps.who.int/iris/bitstream/handle/10665/344424/9789240033986-eng.pdf).

## Discussion

The prevalence, treatment, and control of hypertension vary substantially between different countries with the greatest burden of disease due to hypertension and the lowest proportions of treated and controlled hypertension being in low- and middle-income countries.^16^ Hypertension has been repeatedly demonstrated as the most important single modifiable risk factor responsible for CVD burden of disease and all-cause mortality and hypertension is a significant global public health issue by the WHO.^17^ The global burden of hypertension has been growing over time, driven by changes in lifestyle, population growth, inadequate treatment, and aging. Long-term sequelae of untreated/uncontrolled hypertension include increased CVD events, end organ damage, and mortality.^18,19^ High BP is also closely related to other adverse outcomes including CKD and cognitive impairment/dementia.^2,20^ A wide array of effective hypertension treatment options exists, ranging from lifestyle modifications to various forms of antihypertension medications. Alarmingly, SBP/DBP is controlled to <140/90 mm Hg in <14% of adults worldwide and <8% on low- and middle-income countries.^16^ In the guidelines summarized in Figure 3, the WHO provides the most current and relevant evidence-based guidance for the pharmacological treatment of nonpregnant adults with hypertension. The recommendations pertain to adults with an accurate diagnosis of hypertension who have already received lifestyle counseling. The guidelines provide recommendations for a BP threshold to initiate pharmacological therapy, BP treatment targets, intervals for follow-up visits, and best use of health care workers during treatment of hypertension. The guidelines provide guidance for monotherapy, dual therapy, treatment with single-pill combinations, and the use of treatment algorithms for hypertension management. The recommendations are guided by the quality of the underlying evidence, the balance between expected beneficial and undesirable effects, resource requirements and cost-effectiveness, health equity, acceptability, patient values and preferences, and feasibility of the treatment.

In the GDG recommended guidelines (Figure 3), the evidence base for recommendation on target BP consisted of systematic reviews as well as a review of relevant trials.^21–23^ In patients with comorbidity (CAD, DM, CKD), there is consistent benefit with lower targets (variable thresholds); however, data in these subgroups were imprecise and the evidence was less certain. Therefore, the GDG cautions against applying this evidence to lower-risk patients with raised BP or hypertension. Adverse events such as dizziness in intensive control group and ischemia in patients with coronary artery disease can shift the balance of benefits and harms in older individuals (those aged 65 years or older). Concern about lower adherence due to the need for extra patient and provider effort to reach lower targets should also be balanced against intensive control. The overall certainty of the evidence was judged to be moderate, with large benefits and moderate harms. The GDG made a judgement that the desirable effects outweigh the undesirable effects at a treatment goal of <140/90 mm Hg in all patients with hypertension without comorbidities and SBP <130 mm Hg in high-risk patients with hypertension—those with high CVD risk, diabetes, and CKD. More evidence is required about treatment of those in the SBP 130 to 139 range who fall into one or more of the following subgroups: diabetes, CKD, heart failure, 65 years or older.

CVD risk assessment strategy for those without existing CVD can be based on age, sex, body mass index, BP, previous antihypertensive treatment, smoking, diabetes, and history of CVD.^24^

Most patients with an average SBP ≥140 or DBP ≥90 mm Hg are at high risk for CVD and initiation of antihypertensive drug therapy is indicated. Although helpful, cardiovascular (CVD) risk assessment is not mandatory before initiating antihypertensive drug treatment. CVD risk assessment is most important for guiding decisions about initiating pharmacological treatment for hypertension in those with a lower average SBP (130–139 mm Hg). In all adults with hypertension, it is important that other risk factors for CVD be identified and treated appropriately to lower total cardiovascular risk. Many CVD risk-assessment instruments are available. In the absence of a calibrated equation for the local population, the choice should depend on the resources available, and the acceptability and feasibility of the available CVD risk predicting tools. Whenever CVD risk assessment may impede the timely initiation of hypertension treatment and/or patient follow-up, it should be postponed and included as a follow-up strategy.

Prevailing concepts about BP have changed dramatically over time. Results of the Framingham Heart Study and many other cohort studies have shifted the emphasis from DBP to SBP by demonstrating that SBP was a more important predictor of CVD risk compared with DBP.^25^ In addition, almost all the recent landmark clinical trials have employed SBP rather than DBP as an inclusion criterion and for the trial BP treatment goals. Current US guidelines recommend an intensive BP target of <130/80 mm Hg without mentioning the lower limits of diastolic BP.^26^ A recent cohort study found that lowering diastolic BP to <60 mm Hg was associated with increased risk of cardiovascular events in patients with high cardiovascular risk and a treated SBP <130 mm Hg. The finding that a diastolic BP value between 70- and 80-mm Hg was an optimum target for this patient population merits further study, the results in this DBP group should be interpreted with caution, and these results may not apply to the general population.^27^ Since most recent clinical trials/studies use the SBP to initiate treatment as well as a treatment goal therefore the GDG felt it was appropriate to use SBP in the recommendation.

**Figure 4.**
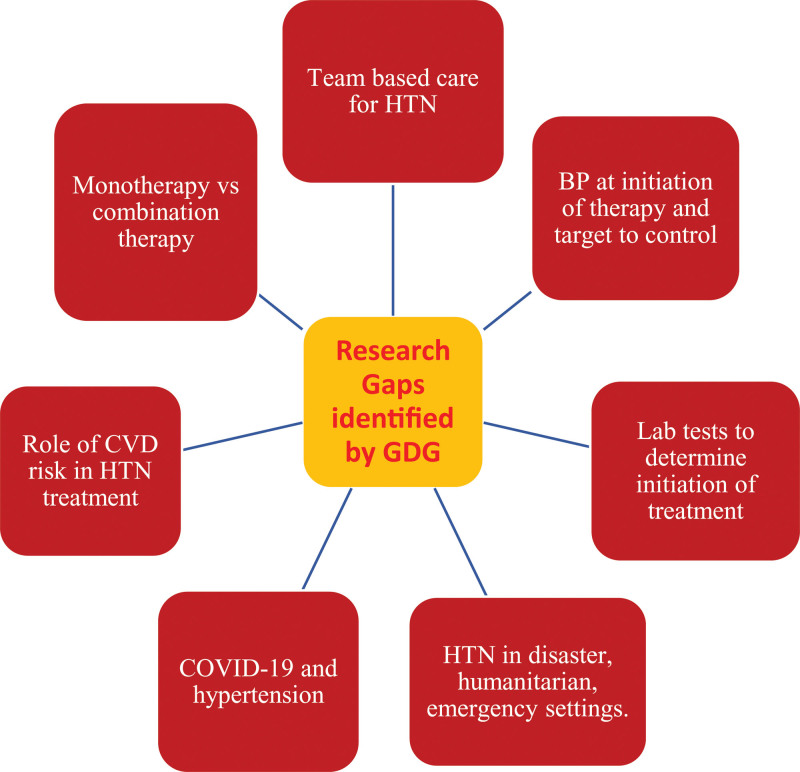
**Research gaps identified by Guideline Development Group (GDG) according to population, intervention, comparison, and outcome.** BP indicates blood pressure; COVID-19, coronavirus disease 2019; CVD, cardiovascular disease; and HTN, hypertension.

Intensive treatment for selected patients adds complexity for health workers; emphasis on team-based care in low-resource settings means that simple, protocolized care is needed. Intensive treatment for some patients complicates treatment protocols and may lead to decisional overload, especially for health workers with more limited training and/or autonomy. On the other hand, strict BP targets in the general population with hypertension are likely to be less acceptable to stakeholders. Most available evidence is derived from high-risk patients receiving intensive treatment and not the general population living with hypertension. Treating BP will reduce health inequity because preventing CV events reduces mortality across the population. Uncontrolled hypertension might be over-represented in vulnerable populations. Therefore, improvement of hypertension treatment and control through better treatment and a lower BP target could reduce long-standing inequality. The goal of the guideline is to facilitate uptake of a standard approach to the pharmacological treatment and management of hypertension which, in turn, will increase the hypertension control rate world-wide. However, several research gaps were identified by the GDG according to the theme of the PICOs (Figure 4). These gaps are summarized as follows:

Thresholds to determine initiation of therapy and targets to achieve BP control: more evidence is required about treatment of those in the baseline SBP 130 to 139 mm Hg range who fall into one or more of the following subgroups: DM, CKD, heart failure, and older age. There is a need for better outcomes data from trials that include dementia and cognitive impairment among outcomes. Clinical significance of treatment-related adverse events registered in clinical trials needs greater clarity. There is a need to quantify the difference in estimates between blinded, placebo-controlled trials and unblinded, active control trials using a standard framework. There is a need for period analysis of trials to capture effects of changes over time in background epidemiology of CVD, non-BP treatments, competing risks, etc. More evidence is needed from low-income countries, middle-income countries, and important high-risk subpopulations living in high-income countries. An assessment of the feasibility, resource needs, and costs of intensive BP treatment of high-risk hypertension patients in real clinical practice is needed. The resource commitment required for more intensive treatment in LMICs needs to be quantified. The opportunity cost of directing resources toward achieving SBP <130 in high-risk individuals needs to be established. Research is needed on the feasibility, acceptability, and efficacy of intensive treatment, especially in high-risk populations in low-income countries and middle-income countries.
Laboratory tests to determine initiation of treatment: a greater understanding is required, of the essential tests to be performed in all patients, to reduce costs and improve outcomes.
Role of CVD risk in hypertension treatment: an exploration is needed of key operational aspects of the implementation of a risk-based approach to CVD prevention and BP-lowering pharmacological treatment in primary health care settings. Additional validation of CVD risk estimating tools, which are known to be population specific, is also needed.
Monotherapy versus combination therapy: a comparison is required of long-term data about hard clinical end points between monotherapy and combination therapy. There is a need for research studies on real-world experiences, designed and statistically powered, to determine if there is a difference in clinical outcomes, such as reduction in MACE, mortality, and serious adverse events between single-pill combinations versus multiple-pill combinations. Health economic analyses are needed to quantify cost-effectiveness and budget implications of implementing incremental initial combination therapy compared with initial monotherapy.
Frequency of reassessment: criteria establishing the clinical definition of stable BP control will be needed to guide the selection of patients requiring less frequent follow-up visits. Research is needed for early and accurate identification of patients less likely to achieve BP control and less likely to follow up as requested by their health care provider. Better evidence is needed on the timing, frequency, and intensity of interventions that improve treatment adherence.
Team-based care for hypertension: evidence is needed that remote monitoring and use of community HCWs/navigators can assist in the management of BP. Evidence of the feasibility, costs, and effectiveness of community/home-based monitoring of BP is needed.
Hypertension in disaster, humanitarian, and emergency settings: assessment of hypertension and appropriate resourcing to treat it should be a priority for agencies providing emergency and longer-term care for patients after or during humanitarian crises to prevent significant mortality and morbidity. Further studies are needed to accurately estimate prevalence of hypertension in crisis-affected populations throughout the world and to evaluate the best treatment approach for this population.
COVID-19 and hypertension: further research that will address key unanswered questions about the role of the RAAS in the pathogenesis and possible treatment of COVID-19 and other coronavirus-based diseases is needed. Prospective studies—in particular, randomized, placebo-controlled trials may provide clearer insight about the effect of ACE inhibitors or ARBs in patients with COVID-19.

## Conclusions

In the above guidelines, the WHO provides the most current and relevant evidence-based global public health guidance on the initiation of treatment with pharmacological agents for hypertension in adults. The guidelines are expected to be valid for a period of 5 years. This period reflects the fact that new research findings are likely to become available in the meantime but also represents a feasible time frame, considering the costs, time and other resources that are needed to update such guidelines. If the evidence base or user needs change before the 5-year mark, consideration will be given to producing updates sooner. More large randomized controlled trials, powered for CVD and/or mortality outcomes, are needed to shed light on the research gaps mentioned in discussion above especially for subpopulations that have not been adequately represented in previous trials.

## Article Information

### Acknowledgments

WHO steering group members: Bernadette Cappello, Neerja Chowdhury, Gampo Dorji, Jill Farrington, Pedro Ordunez, Steven Shongwe, Slim Slama, Cherian Varghese, Marco Vitoria, Temo Waqanivalu. Systematic review team: Abdallah Al Elayhi, Romina Brignardello, Sara Jdiaa, Veena Manja (University of Kansas Medical Center, Kansas). External review group: Jennifer Cohn, Prabhdeep Kaur, Venus Mushininga, Daniel T Lackland, Marcelo Orias, Antoinette Péchère Bertschi, and Xin Hua Zhang. WHO Staff: Bente Mikkelsen, Rebekah Thomas, Nathan Ford, Alma Alic, Sheila Nakpil.

### Sources of Funding

The development of this guideline was financially supported by the US Centers for Disease Control and Prevention and the World Health Organization.

### Disclosures

None.
